# Effect of Ammonium Polyphosphate to Aluminum Hydroxide Mass Ratio on the Properties of Wood-Flour/Polypropylene Composites

**DOI:** 10.3390/polym9110615

**Published:** 2017-11-14

**Authors:** Wen Wang, Yao Peng, Mauro Zammarano, Wei Zhang, Jianzhang Li

**Affiliations:** 1 Ministry of Education Key Laboratory of Wood Material Science and Utilization, Beijing Forestry University, Beijing 100083, China; wangw-1990@163.com (W.W.); pengyao0304@163.com (Y.P.); zhangweishe@126.com (W.Z.); 2 Beijing Key Laboratory of Wood Science and Engineering, Beijing Forestry University, Beijing 100083, China; 3 Fire Research Division, Engineering Laboratory, National Institute of Standards and Technology, Gaithersburg, MD 20899, USA; mauro.zammarano@nist.gov; 4 Faculty of Forestry, University of Toronto, Ontario, ON M5S 3B3, Canada

**Keywords:** wood-plastic composite, ammonium polypropylene, aluminum hydroxide, physical-mechanical property, fire performance, smoke generation, degree of graphitization

## Abstract

Two halogen-free inorganic flame retardants, ammonium polyphosphate (APP) and aluminum hydroxide (ATH) were added to wood-flour/polypropylene composites (WPCs) at different APP to ATH mass ratios (APP/ATH ratios), with a constant total loading of 30 wt % (30% by mass). Water soaking tests indicated a low hygroscopicity and/or solubility of ATH as compared to APP. Mechanical property tests showed that the flexural properties were not significantly affected by the APP/ATH ratio, while the impact strength appeared to increase with the increasing ATH/APP ratio. Cone calorimetry indicated that APP appeared to be more effective than ATH in reducing the peak of heat release rate (PHRR). However, when compared to the neat WPCs, total smoke release decreased with the addition of ATH but increased with the addition of APP. Noticeably, WPCs containing the combination of 20 wt % APP and 10 wt % ATH (WPC/APP-20/ATH-10) showed the lowest PHRR and total heat release in all of the formulations. WPCs combustion residues were analyzed by scanning electron microscopy, laser Raman spectroscopy, and Fourier transform infrared spectroscopy (FTIR). Thermogravimetric analysis coupled with FTIR spectroscopy was used to identify the organic volatiles that were produced during the thermal decomposition of WPCs. WPC/APP-20/ATH-10 showed the most compact carbonaceous residue with the highest degree of graphitization.

## 1. Introduction

Wood-plastic composites (WPCs), consisting of wood processing residue (as fibers or finely ground particles) and thermoplastic polymer, have been showing a rapid growing usage in the last decades due to their significant advantages, such as high durability, low maintenance and cost, relatively high strength and stiffness, sustainability, and wood-like material appearance and properties [1,2,3,4]. WPCs have been widely used in both exterior and interior applications, including decking, fencing, cladding, window and door frames, landscaping timbers, and automotive components, etc. [5,6,7,8]. However, the high flammability of WPCs may limit their application. Several researchers have focused on the method to improve the flame retardancy of WPCs [9,10,11,12,13,14,15]. 

The most widely used method is to incorporate flame retardants (FRs) directly into WPCs during the melt processing [16,17,18,19,20]. Halogenated compounds based on chlorine or bromine are effective FRs for polymers and composites. However, their use is declining due to potential environmental and safety issues. For example, harmful gases can be generated during the combustion of halogenated compounds and this can make handling and disposal problems critical [21]. As a consequence, the market is looking for halogen-free FRs with a benign toxicological profile. Ammonium polyphosphate (APP) is a commonly used halogen-free FR for wood [22], polymers [23,24,25,26,27], as well as WPCs [28,29,30,31,32] due to its high efficiency and low toxicity. The addition of APP promotes the formation of an intumescent char layer that acts as a physical barrier to slow the heat and mass transfer through the surface of the material. However, the low hydrolytic stability of APP and the low compatibility between APP and the thermoplastic matrix have a negative effect on the durability and physical-mechanical properties of the composites [28,30,31,32]. Moreover, APP could also increase the smoke generation of the matrix during combustion [27,32,33]. 

Aluminum hydroxide (ATH) is a low-cost halogen-free FR, which is widely used in polymers. During its endothermic decomposition process, it will withdraw heat from surroundings, hence retarding the decomposition of the substrate [34]. Water is also released and dilutes the combustible volatiles that are produced by the polymer. Furthermore, ATH decomposes to Al_2_O_3_, which accumulates on the surface of the sample and protects the substrate by impeding heat and mass transfer [35]. Generally, ATH serves a threefold purpose of filler, FR, and smoke suppressant in polymeric materials [36,37]. However, the high loading of ATH (approximately 60 wt %) is usually required to provide an adequate flame retardancy that inevitably has a detrimental effect on the mechanical properties and processing of the composites [37,38,39]. These problems can be mitigated by incorporating other FRs and reducing the total FR content [40,41].

The effect of combining ATH and APP is controversial and might be affected by the specific polymer system, ATH-to-APP ratio, viscosity of the decomposition products, and fire scenario. For example, an antagonistic effect in the styrene butadiene rubber has been reported by cone calorimetry tests (heat flux was 30 kW/m^2^) in terms of peak of heat release rate (PHRR) and total heat release, due to the formation of aluminum orthophosphate and aluminum metaphosphate, and the reduction in ultraphosphate generation [42]. However, another study reported that aluminum metaphosphate acted as an effective thermal barrier in an intumescent flame-retardant polypropylene formulation. The replacement of 2 wt % APP-dipentaerythritol FR system (25 wt %, APP/dipentaerythritol mass ratio = 2.5) with nano-ATH increased the limiting oxygen index by 17%, improved the UL-94 rate from V-1 to V-0, and increased PHRR value in cone calorimetry (heat flux was 50 kW/m^2^) by 57% as well [43]. 

In intumescent systems, the proper tuning of the viscosity for liquefied material (produced by thermal decomposition) plays a key role [44,45,46]. The combination of inorganic hydroxide, like ATH and APP, might prevent the collapse of the foamed structure in intumescent system by increasing the viscosity of the liquefied material. Therefore, the viscosity of the intumescent system might be adjusted by varying the APP to ATH mass ratio [47]. The aim of this work was to investigate different APP to ATH mass ratios on the physical-mechanical properties, thermal behavior, and flame retardant performance of wood-flour/polypropylene composites, as well as to provide an insight into the flame-retardant mechanism of APP and ATH combination system in WPCs.

## 2. Materials and Methods

### 2.1. Materials

Wood-flour (WF, *Populus tomentasa* Carr., particle size between 60 and 80 mesh) was ground from wood processing residue and was kindly provided by Hebei Gaocheng Xingda Wood Flour Company, Gaocheng, China. Polypropylene (PP, K-8303, copolymer, melt-flow rate 1.5 g/10 min) was purchased from Beijing Yanshan Petrochemical Co. Ltd., Beijing, China. Maleic anhydride-grafted polypropylene (PP-*g*-MAH, H9522, melt-flow rate 120 g/10 min) was purchased from Ningbo Kyler Trading Co. Ltd., Ningbo, China. Ammonium polyphosphate (APP, phase II, degree of polymerization >1000, average particle size ≈ 10 μm) was obtained from Shandong Shian Chemical Co. Ltd., Dezhou, China. Aluminum hydroxide (ATH, average particle size ≈ 10 μm) was kindly supplied by Zhongzhou Branch Company of China Aluminum Co. Ltd., Jiaozuo, China.

### 2.2. Preparation of the Flame-Retardant Composites

WF was dried in an oven at 80 °C for 1 day. PP, PP-*g*-MAH, APP, and ATH were also dried at 80 °C for 12 h and then they were mixed together with the WF using a high-intensity blender (SRH-10, Beijing Zedao Co. Ltd., Beijing, China) at 2900 rotations/min for 4 min at room temperature. The polymer/WF mass ratio and FR loading for all of the WPCs were the same (3/2 and 30 wt %, respectively). The composition of each WPC is shown in Table 1. After the mixtures were dried, they were melt-blended in a co-rotating twin-screw extruder (KESUN KS-20, Kunshan Rubber and Plastic Machinery Ltd., Kunshan, China). Generally, the extrusion temperature of PP is about 20 °C higher than its melting temperature (165–170 °C). However, WF starts decomposing above 180 °C. Therefore, in this study, the corresponding temperatures in the extruder barrel were controlled at 165 °C, 170 °C, 175 °C, 180 °C, and 175 °C from hopper to die zones, respectively. The screw speed was 167 rotations/min. Then, the extrudated materials were pelletized before being used for hand matting. Composite plates with the dimension of 270 mm × 270 mm × 3 mm were produced through hot and cold pressing, following the methods reported in our previous literature [31]. Samples were prepared by cutting the composite plates to the dimensions required by the tests. 

### 2.3. Physical-Mechanical Properties

#### 2.3.1. Water Soaking Test

The test specimens were immersed into distilled water at (25 ± 2) °C for 15 days. Then the water absorption (WA) values were calculated based on the weight changes after 6 h, 24 h, and thereafter at 48 h intervals by removing the surface water with a dry cloth. Six replicates (50 mm × 50 mm × 3 mm) were tested for each group. The values of WA as percentages were calculated with the following equation:

*WA* (%) = ((*W_t_* − *W*_0_)/*W*_0_) × 100%

where *W*_0_ is the weight of oven-dried sample at 60 °C for 12 h before immersion and *W_t_* is the weight of the sample at a given immersion time *t*.

#### 2.3.2. Three-Point Bending Test

Three-point tests were performed at a cross-head speed of 5 mm/min in accordance with the Chinese standard GB/T 9341-2008 (equivalent to ISO 178-2001). Six replicates (60 mm × 25 mm × 3 mm) were tested for each group.

#### 2.3.3. Impact Strength

Unnotched Izod impact tests were carried out in accordance with the Chinese standard GB/T 1843-2008 (equivalent to ISO 180-2000). Six unnotched replicates (80 mm × 10 mm × 3 mm) were tested for each group. 

### 2.4. Thermogravimetric Analysis (TGA)

The thermal stability of WPCs was evaluated by TGA (Q50 TGA analyzer, TA Instruments Ltd., New Castle, DE, USA). Each formulation (about 7 mg) was tested under a nitrogen flow (40 mL/min) at a linear heating rate of 20 °C/min from room temperature up to 700 °C.

### 2.5. Cone Calorimetry

Cone calorimetry (Fire Testing Technology Ltd., East Grinstead, UK) was performed at an incident heat flux of 50 kW/m^2^ in accordance with ISO 5660 standard procedures. Each specimen (100 mm × 100 mm × 3 mm) was wrapped in aluminum foil. Three replicates were tested for each group.

### 2.6. Scanning Electron Microscopy (SEM)

The surface morphology of WPCs combustion residues was investigated by a field-emission SU8010 SEM analyzer (Hitachi Ltd., Tokyo, Japan) with an acceleration voltage of 3.0 kV. All of the samples were sputter-coated with gold layer. 

### 2.7. Laser Raman Spectroscopy (LRS)

LRS spectra of WPCs combustion residues at the end of cone calorimetry tests were investigated by a SPEX-1403 spectrometer (SPEX SamplePrep Ltd., Metuchen, NJ, USA). The spectra were recorded under an Ar-ion laser (514.5 nm) at room temperature. Each spectrum was subjected to peak fitting using the software Origin 9.0/Multiple Peak Fit Module to resolve the curve into two Lorentz bands.

### 2.8. Fourier Transform Infrared Spectroscopy (FTIR)

FTIR spectra of WPCs combustion residues at the end of cone calorimetry tests were recorded by a Nicolet 6700 FTIR spectrometer (Thermo Fisher Scientific Ltd., Waltham, MA, USA) in attenuated total reflection mode. Before testing, all the samples were ground into powders and oven dried. Spectra were signal averaged over 32 scans at a resolution of 4 cm^−1^ in the (4000–650) cm^−1^ wavenumber region. 

### 2.9. Thermogravimetric Analysis-Infrared Spectrometry (TGA-IR)

TGA-IR spectra were recorded using a Q 5000 TGA analyzer (TA Instruments Ltd., New Castle, DE, USA), which was interfaced to a Nicolet 6700 FTIR spectrophotometer (Thermo Fisher Scientific Ltd., Waltham, MA, USA). Samples (about 15 mg) were tested in an alumina crucible at a 45 mL/min nitrogen flow and at a heating rate of 20 °C/min from room temperature up to 700 °C.

## 3. Results and Discussion

### 3.1. Physical-Mechanical Performance of WPCs

**Figure 1:**
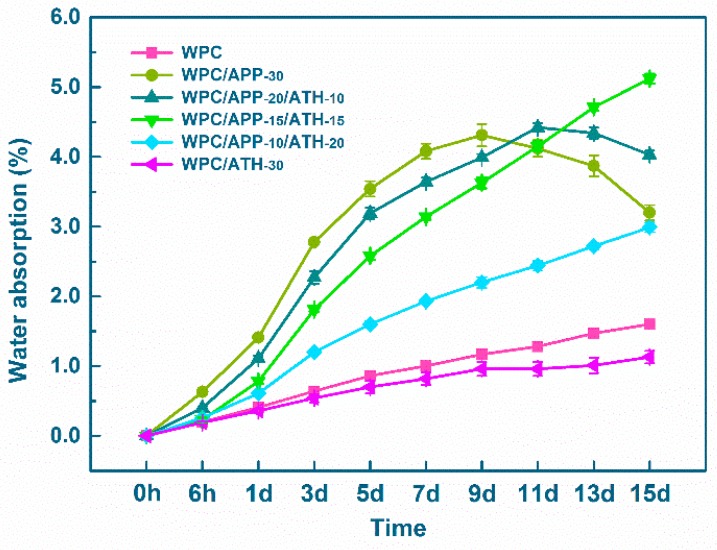
Water absorption (WA) values of WPCs (error bars shown here are equal to ±one standard deviation).

As can be seen in Figure 1, WA of the neat WPCs (no FR fillers) increased at a constant rate in the investigated time range. This was attributed to the slow diffusion of water molecules into the WF/polymer interface, likely promoting by hydrogen bonding between the water molecules and the free –OH groups in the WF [48]. When 30 wt % APP was added, WA value of the composites (WPC/APP-30) dramatically increased because of the high hygroscopicity of APP. However, the WA value showed a maximum at the ninth day, and then decreased significantly, likely due to partial APP dissolution in water [31]. When 30 wt % ATH was added, WA value of the composites (WPC/ATH-30) slightly but obviously decreased as compared to the neat WPC throughout the entire tested time range. The WPC/ATH-30 showed the lowest WA value, even lower than the neat WPCs. When compared with the WPC/APP-30, WA value of the composites containing ATH decreased with the increase of ATH loading in the matrix. The maximum in WA occurred at the 11th day of immersion for WPC/APP-20/ATH-10, two days after the maximum observed for WPC/APP-30. It should be noted that the WA maxima were not observed for the WPC/APP-15/ATH-15, WPC/APP-10/ATH-20, and WPC/ATH-30 within the tested 15 days. According to the results, APP increased the hygroscopicity and limited the hydrolytic stability of the composites. A significant amount of APP was solubilized and extracted from the composites over the tested 15 days. On the other side, ATH appeared to decrease the hygroscopicity of WPCs and increase the hydrolytic stability of the composites as compared to APP.

**Figure 2:**
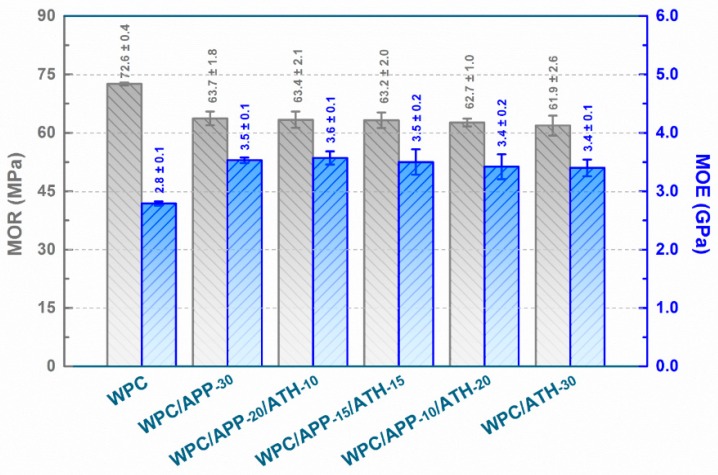
Flexural strength (MOR) and flexural modulus (MOE) values of WPCs (error bars shown here are equal to ±one standard deviation).

When the composites are used as load bearing structures in furniture, automotive, or building industries, their flexural strength (MOR) and flexural modulus (MOE) are the critical performance metrics [49]. Figure 2 shows that the incorporation of 30 wt % APP significantly decreased the MOR of the composites from (72.6 ± 0.4) MPa to (63.7 ± 1.8) MPa (≈12% average decrease), likely due to the poor APP/matrix compatibility, and increased the MOE from (2.8 ± 0.1) GPa to (3.5 ± 0.1) GPa (≈26% average increase) due to the intrinsic high MOE of APP. These results were consistent with the previous reports [31,34,50,51]. ATH had a similar effect on the MOR and MOE. The incorporation of 30 wt % ATH decreased the MOR of composites to (61.9 ± 2.6) MPa (≈15% average decrease) and increased the MOE of composites to (3.4 ± 0.1) GPa (≈22% average increase). The MOR and MOE values were not significantly affected by the APP to ATH mass ratio (see WPC/APP-20/ATH-10, WPC/APP-15/ATH-15, WPC/APP-10/ATH-20 in Figure 2).

**Figure 3:**
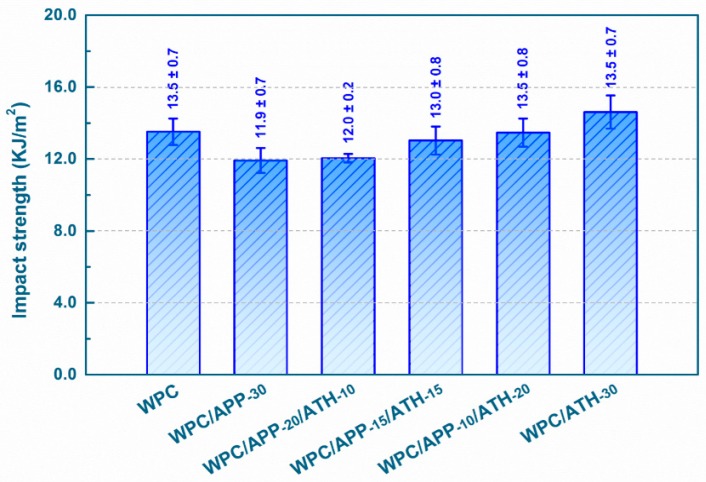
Impact strength of WPCs (error bars shown here are equal to ±one standard deviation).

The results of impact strength tests are shown in Figure 3. As compared to the neat WPCs without FR fillers, the impact strength decreased from (13.5 ± 0.7) KJ/m^2^ to (11.9 ± 0.7) KJ/m^2^ (about 12% average decrease) with the addition of 30 wt % APP. This might be due to the presence of residual agglomerates of APP, which act as micro-crack initiators [31,52]. As more APP was replaced by ATH in the composites (see WPC/APP-20/ATH-10, WPC/APP-15/ATH-15, and WPC/APP-10/ATH-20 in Figure 3), a gradual increase in impact strength was observed. The impact strength increased up to (14.6 ± 0.9) KJ/m^2^ with the addition of 30 wt % ATH. The improvement in impact strength achieved by replacing APP with ATH could be due to several reasons, such as, a stronger particle-matrix interface, better dispersion, and a variation in the PP crystallinity.

### 3.2. Thermal Degradation Analysis

**Figure 4:**
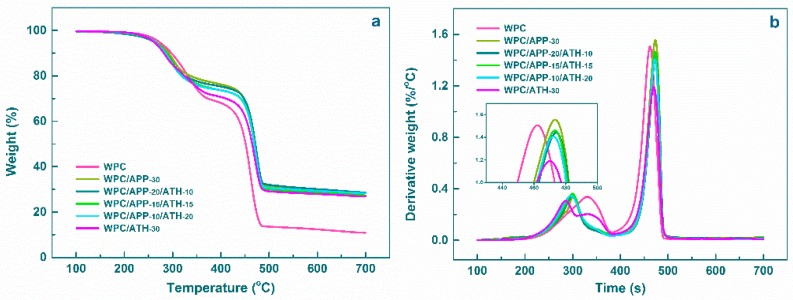
Thermogravimetric Analysis (TGA) (**a**) and derivative thermogravimetry (DTG) (**b**) curves of WPCs.

TGA and derivative thermogravimetry (DTG) curves of the WPCs are displayed in Figure 4a,b, respectively, and the related data are summarized in Table 2. The thermal degradation process of the neat WPCs showed two main degradation steps (DSs), with only 10.8 wt % residue being left at 700 °C. The temperatures at which the derivative weight peaks are achieved for the first DS, the second DS, and the third DS are indicated in Table 2 as *T_max_*_1_, *T_max_*_2_, and *T_max_*_3_, respectively. For the neat WPCs, *T_max_*_1_, and *T_max_*_2_ were 330 °C and 462 °C, respectively. The WPCs containing APP or a combination of APP and ATH showed lower *T_max_*_1_ and higher *T_max_*_2_ than the neat WPCs without APP. The lower *T_max_*_1_ indicated that the presence of APP accelerated the thermal decomposition of the composites. WPCs containing APP (WPC/APP-30, WPC/APP-20/ATH-10, WPC/APP-15/ATH-15, and WPC/APP-10/ATH-20) also showed a higher residue at 700 °C (Table 2) than the neat WPCs due to polymer charring (promoted by APP) and thermally stable aluminum-orthophosphate and aluminum metaphosphate (generated by the reaction between ATH and APP) [42]. Noticeably, WPC/APP-20/ATH-10 was the sample with the highest residue. Unlike other formulations, WPC/ATH-30 showed three different DSs. The first DS was mainly due to the thermal degradation of ATH [43]. The other two DSs were similar to those of the neat WPCs. ATH decomposed without significantly affecting the decomposition of the WPCs and without promoting charring reactions. As a result, WPC/ATH-30 generated less residue than the other WPCs containing FR fillers.

### 3.3. Cone Calorimetry

**Figure 5:**
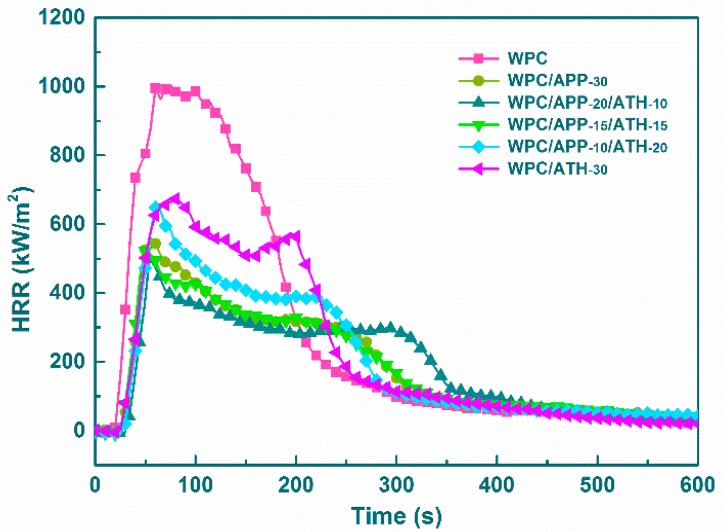
Heat release rate (HRR) curves of WPCs.

Cone calorimetry was used to assess the forced combustion behavior of WPCs. Table 3 lists the corresponding data, including the ignition time (IT), peak heat release rate (PHRR), average heat release rate (AHRR), total heat release (THR), and residue. Heat release rate (HRR) is the most important parameter for evaluating the flame retardancy [53]. The HRR curves of WPCs are displayed in Figure 5. As can be observed, the neat WPCs without FR fillers showed the highest HRR with a prolonged PHRR of about 1000 kW/m^2^ between 60 s and 120 s. It yielded a short IT of (15 ± 2) s, a THR of (170 ± 3) MJ/m^2^, and a residue of (6 ± 1) wt %. The addition of 30 wt % APP significantly delayed the IT to (23 ± 2) s, decreased the PHRR, AHRR, and THR to (560 ± 3) kW/m^2^, (164 ± 2) kW/m^2^, and (124 ± 2) MJ/m^2^, respectively, and increased the residue at the end of the tests to (19 ± 1) wt %. These results agreed with previously published reports [28,31,32]. When 10 wt % APP was replaced by ATH, a further improvement in performance was observed. The IT increased to (29 ± 2) s, the PHRR and THR decreased to (498 ± 3) kW/m^2^ and (115 ± 2) MJ/m^2^, respectively, and the residue increased to (27 ± 2) wt %. This high residue (consistent with the TGA results) could act as an effective barrier layer on the sample surface as a result of the reaction between ATH and APP to generate a thermally stable aluminum-orthophosphate and aluminum-metaphosphate [42]. WPC/APP-20/ATH-10 showed the best flame-retardant performance between the WPCs investigated in this study. A decrease in performance was observed when more APP was replaced by ATH.

**Figure 6:**
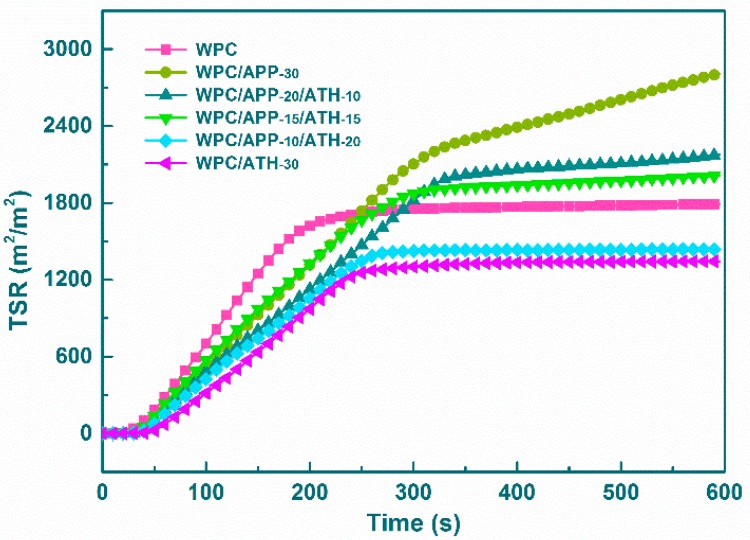
Total smoke release (TSR) curves of WPCs.

The evolution of smoke is considered to be another important parameter for flame-retardant materials. Figure 6 presents the total smoke release (TSR) curves of WPCs. The related smoke property data are summarized in Table 4, including the yield of carbon monoxide (*Y*co), yield of carbon dioxide (*Y*co_2_), CO/CO_2_ weight ratio, and TSR. The higher this ratio, the greater the toxicity of the produced combustion products [54]. The incorporation of 30 wt % APP into WPCs significantly increased the TSR and CO/CO_2_ weight ratio by 57% and 387%, respectively. These two parameters continued to reduce when APP was replaced by more and more ATH. Noticeably, WPC/ATH-30 had an even lower TSR and CO/CO_2_ weight ratio than the neat WPCs without FR fillers. This is due to the release of water vapor (generated by ATH decomposition) that diluted the combustion products and inhibited soot formation in the gas phase [55].

### 3.4. Residue Morphology and Structure

**Figure 7:**
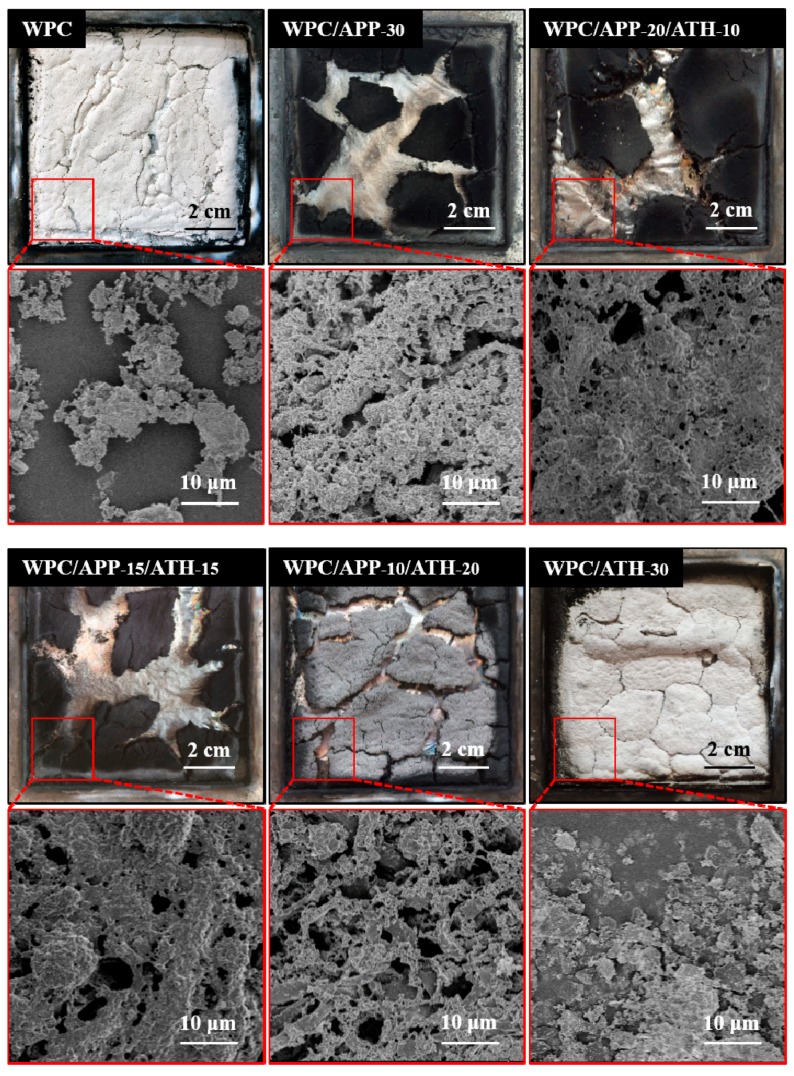
Surface morphologies of the residues generated by WPCs after cone calorimetry.

Figure 7 shows the optical microscopy and SEM micrographs of the residues after cone calorimetry. The white color observed in the neat WPCs indicated the formation of ash and the absence of substantial carbonaceous residue on the surface of the sample. The WPC/APP-30 residue showed an expanded honeycomb-like structure with multiple cracks and holes on the surface. This loose char structure was not effective enough to act as a heat insulating barrier at high temperatures [56]. In comparison, WPC/APP-20/ATH-10 had a more compact and smoother carbonaceous residue (less holes and cracks on the surface) that can likely decrease the burning rate by generating a more effective heat and mass transfer on the surface of the sample. Therefore, WPC/APP-20/ATH-10 showed a lower PHRR and THR values than WPC/APP-30, as discussed above (Table 3). When more APP was replaced by ATH, the carbonaceous residues of WPCs became looser and less compact, with more holes and cracks on the surface. Finally, as indicated by the white color of the residue, WPC/ATH-30 showed the generation of an effective inorganic barrier without significant charring (as expected due to the absence of APP acting as charring agent).

Raman spectroscopy has been applied to study the char structure and to further investigate the flame-retardant mechanism [57,58]. The D band (at around 1585 cm^−1^) is associated to the presence of amorphous carbon, whereas the G band (at around 1360 cm^−1^) is associated to the presence of graphitic carbon [59,60]. The integrated area ratio of the D band and G band (*I_D_*/*I_G_*) can be used to estimate the graphitization degree of the carbonaceous char (*I_D_*/*I_G_* ratio is inversely proportional to the degree of graphitization). Graphitic carbon is more thermally stable than amorphous carbon, so the higher the degree of graphitization, the more durable and effective the barrier effect (i.e., effectively prevent the heat and mass transfer between the burning matrix surface and the underlying material) of the char [58,61].

**Figure 8:**
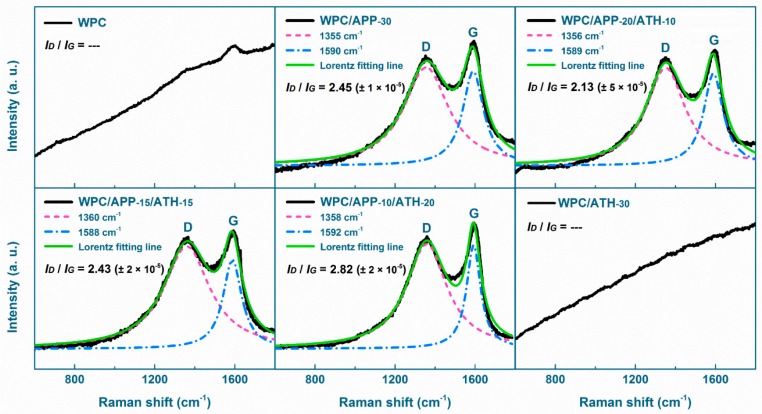
Raman spectra of WPCs combustion residues.

Figure 8 presents the Raman spectra of WPCs combustion residues after cone calorimetry. As can be seen in Figure 8, the *I_D_*/*I_G_* ratio values are 2.13, 2.43, 2.45, 2.82 for WPC/APP-20/ATH-10, WPC/APP-15/ATH-15, WPC/APP-30, WPC/APP-10/ATH-20, respectively. These data indicated that the combination of 20 wt % APP and 10 wt % ATH provided the WPCs with the highest degree of graphitization and the most thermally stable char structure. These results agree with the TGA and cone calorimetry tests, where WPC/APP-20/ATH-10 showed the highest residue, and might suggest that the level of graphitization plays a significant role in the flame-retardant mechanism.

**Figure 9:**
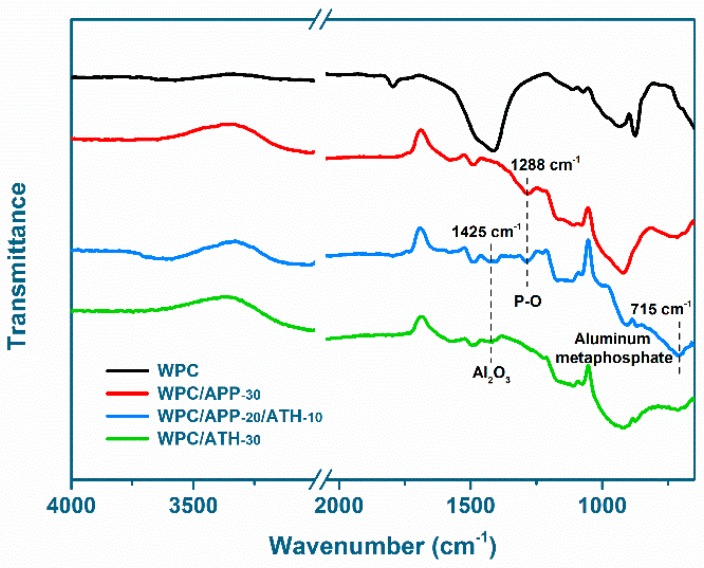
Fourier transform infrared spectroscopy (FTIR) spectra of WPCs combustion residues.

To further understand the flame-retardant mechanism of APP and ATH in WPCs, the cone calorimeter residues were investigated by FTIR (Figure 9). The major absorption peaks of the neat WPCs were at 1795 cm^−1^ (C=O stretching), 1410 cm^−1^ (O–H bending), and the region between 1120 cm^−1^ to 650 cm^−1^ (C–H bending) [32]. The residues of the flame-retardant WPCs showed three additional features in the FTIR spectra. The first one is the presence of an absorption peak for WPC/APP-30 and WPC/APP-20/ATH-10 at 1288 cm^−1^, which was attributed to the P–O stretching of decomposed APP. The second one is the presence of an absorption peak for WPC/APP-20/ATH-10 and WPC/ATH-30 at 1425 cm^−1^, which was likely due to the Al_2_O_3_ produced by ATH decomposition. The last one is the absorption peak at about 715 cm^−1^, which is a characteristic peak of aluminum metaphosphate [62]. This peak is obvious in WPC/APP-20/ATH-10, indicating that APP and ATH underwent a chemical reaction during the thermal decomposition process to generate aluminum metaphosphate. The formed aluminum metaphosphate had an ultra-high thermal stability [43] that could enhance the residue and barrier effect during the combustion process.

### 3.5. Evolved Gas Analysis

**Figure 10:**
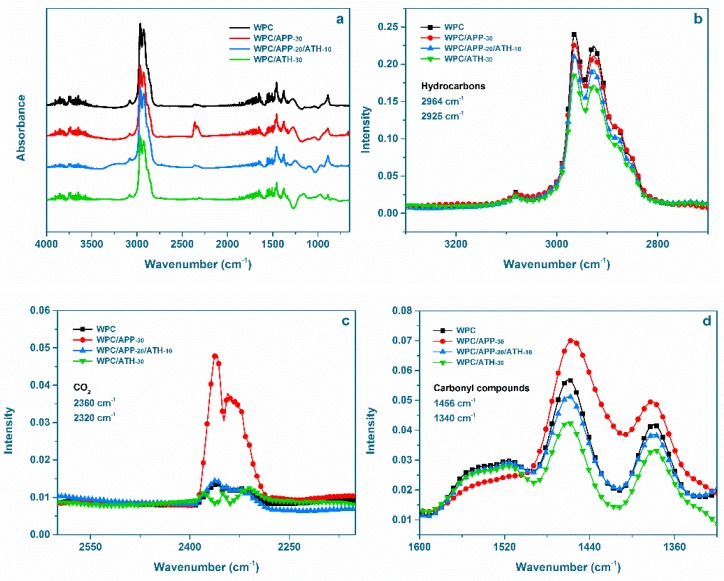
(**a**) FTIR spectra of volatilized pyrolysis products evolved by anaerobic pyrolysis of WPCs at the maximum of mass loss rate; the absorption intensities of the characteristic peaks of (**b**) hydrocarbons, (**c**) CO_2_, and (**d**) carbonyl compounds.

The volatiles produced during the anaerobic pyrolysis of WPCs were investigated by TGA-IR. Figure 10a displays the FTIR spectra at the maximum of mass loss rate. The FTIR characteristic peaks of the volatilized gases in the spectra agree with previously reported data and are assigned to the generation of H_2_O (3800 cm^−1^ to 3200 cm^−1^), hydrocarbons (2800 cm^−1^ to 3000 cm^−1^), CO_2_ (2340 cm^−1^), and different carbonyl compounds (1750 cm^−1^ to 650 cm^−1^) [61]. The intensities of the characteristic absorption for hydrocarbons, CO_2_, and carbonyl compounds are shown in Figure 10b–d, respectively. As can be seen, the absorption intensity for hydrocarbons, CO_2_, and carbonyl compounds increased with the addition of APP and decreased with the addition of ATH in WPCs. A decrease in the generation rate of organic volatiles implies that there is less “fuel” available to feed flaming combustion at the peak of mass lose rate, thus, the PHRR might be reduced [61]. TGA-IR data clearly show the smoke suppression effect of ATH during combustion. The reduction of hydrocarbons can in fact suppress soot generation [63]. These data agree with the measured low TSR value and SDR value for the ATH-containing WPCs in the cone calorimetry and the smoke density tests, respectively.

## 4. Conclusions

In this study, different APP to ATH mass ratios were incorporated into wood-flour/polypropylene composites (WPCs). Water soaking tests indicated that the replacement of APP with ATH reduced the water absorption of WPCs, likely due to the decreased hygroscopicity. The flexural properties were not significantly affected by the APP to ATH mass ratio. The addition of 30 wt % APP and/or ATH decreased the flexural strength of the neat WPCs (no flame retardants) by about 15% and increased the flexural modulus by about 25%. The impact strength appeared to increase with the APP to ATH mass ratio decrease. No reduction in impact strength was observed in the WPCs containing 30 wt % ATH as compared to the neat WPCs. Cone calorimetry data showed that the partial replacement of APP with ATH decreased the heat release rate and smoke generation during combustion. Among all of the WPCs investigated here, WPC/APP-20/ATH-10 showed the lowest PHRR and THR values with a reduction of about 50% and 32%, respectively, as compared to the neat WPCs. WPC/APP-20/ATH-10 was also the sample with the best char (the highest residue with the most compact carbonaceous structure and the highest degree of graphitization) that could act as a physical barrier to protect the underlying matrix. Therefore, the formation of an effective physical barrier was likely playing a key role in terms of fire behavior. In conclusion, the data showed that the combination of APP and ATH at a proper mass ratio had a beneficial effect on smoke and heat generation of WPCs during forced combustion.

## Figures and Tables

**Table 1:** Compositions of the wood-plastic composites (WPCs).

Samples	PP (wt %)	WF (wt %)	PP-*g*-MAH (wt %)	APP (wt %)	ATH (wt %)
WPC	55	40	5	-	-
WPC/APP-30	37	28	5	30	-
WPC/APP-20/ATH-10	37	28	5	20	10
WPC/APP-15/ATH-15	37	28	5	15	15
WPC/APP-10/ATH-20	37	28	5	10	20
WPC/ATH-30	37	28	5	-	30

**Table 2:** TGA data of WPCs.

Samples	*T_max_*_1_^a^ (°C)	*T_max_*_2_^b^ (°C)	*T_max_*_3_^c^ (°C)	Residue (wt %)
WPC	330	462	-	10.8
WPC/APP-30	303	473	-	27.3
WPC/APP-20/ATH-10	299	474	-	28.6
WPC/APP-15/ATH-15	300	473	-	28.2
WPC/APP-10/ATH-20	296	472	-	27.3
WPC/ATH-30	285	331	470	26.8

^a^ Temperature at which the first peak of derivative weight occurred; ^b^ Temperature at which the second peak of derivative weight occurred; ^c^ Temperature at which the third peak of derivative weight occurred.

**Table 3:** Cone calorimetry test parameters of WPCs.

Samples	IT	PHRR	AHRR	THR	Residue
	(s)	(kW/m^2^)	(kW/m^2^)	(MJ/m^2^)	(wt %)
WPC	15 ± 2	1003 ± 3	292 ± 4	170 ± 3	6 ± 1
WPC/APP-30	23 ± 2	560 ± 3	164 ± 2	124 ± 2	19 ± 1
WPC/APP-20/ATH-10	29 ± 2	498 ± 3	164 ± 2	115 ± 2	27 ± 2
WPC/APP-15/ATH-15	24 ± 2	522 ± 4	162 ± 1	119 ± 3	25 ± 1
WPC/APP-10/ATH-20	24 ± 1	595 ± 3	173 ± 3	128 ± 2	25 ± 2
WPC/ATH-30	24 ± 2	673 ± 2	184 ± 3	136 ± 1	22 ± 1

**Table 4:** Smoke generation data of WPCs measured by cone calorimetry.

Samples	*Y*co	*Y*co_2_	CO/CO_2_	TSR
	(kg/kg)	(kg/kg)	Weight Ratio	(m^2^/m^2^)
WPC	0.071 ± 0.001	3.91 ± 0.01	0.018 ± 0.001	1789 ± 11
WPC/APP-30	0.227 ± 0.007	2.56 ± 0.02	0.089 ± 0.003	2808 ± 15
WPC/APP-20/ATH-10	0.222 ± 0.018	2.57 ± 0.01	0.086 ± 0.007	2176 ± 16
WPC/APP-15/ATH-15	0.216 ± 0.011	2.75 ± 0.01	0.078 ± 0.004	2012 ± 13
WPC/APP-10/ATH-20	0.103 ± 0.002	3.13 ± 0.03	0.033 ± 0.001	1437 ± 19
WPC/ATH-30	0.053 ± 0.001	3.38 ± 0.02	0.016 ± 0.001	1344 ± 13
